# Effects of NMDA receptor antagonists and antipsychotics on high frequency oscillations recorded in the nucleus accumbens of freely moving mice

**DOI:** 10.1007/s00213-015-4073-0

**Published:** 2015-10-08

**Authors:** Mark J. Hunt, Maciej Olszewski, Joanna Piasecka, Miles A. Whittington, Stefan Kasicki

**Affiliations:** Laboratory of the Limbic System, Nencki Institute of Experimental Biology, 3 Pasteur Street, 02-093 Warsaw, Poland; The Hull York Medical School, University of York, Heslington, York YO10 5DD UK

**Keywords:** NMDA receptor antagonists, Antipsychotics, Oscillations, Mice, Schizophrenia

## Abstract

**Rationale:**

Abnormal oscillatory activity associated with *N*-methyl-D-aspartate (NMDA) receptor hypofunction is widely considered to contribute to the symptoms of schizophrenia.

**Objective:**

This study aims to characterise the changes produced by NMDA receptor antagonists and antipsychotics on accumbal high-frequency oscillations (HFO; 130–180 Hz) in mice.

**Methods:**

Local field potentials were recorded from the nucleus accumbens of freely moving mice.

**Results:**

Systemic injection of ketamine and MK801 both dose-dependently increased the power of HFO and produced small increases in HFO frequency. The atypical antipsychotic drug, clozapine, produced a robust dose-dependent reduction in the frequency of MK801-enhanced HFO, whilst haloperidol, a typical antipsychotic drug, had little effect. Stimulation of NMDA receptors (directly or through the glycine site) as well as activation of 5-HT_1A_ receptors, reduced the frequency of MK801-enhanced HFO, but other receptors known to be targets for clozapine, namely 5-HT_2A_, 5-HT_7_ and histamine H_3_ receptors had no effect.

**Conclusions:**

NMDA receptor antagonists and antipsychotics produce broadly similar fundamental effects on HFO, as reported previously for rats, but we did observe several notable differences. In mice, HFO at baseline were weak or not detectable unlike rats. Post-injection of NMDA receptor antagonists HFO was also weaker but significantly faster. Additionally, we found that atypical antipsychotic drugs may reduce the frequency of HFO by interacting with NMDA and/or 5-HT_1A_ receptors.

**Electronic supplementary material:**

The online version of this article (doi:10.1007/s00213-015-4073-0) contains supplementary material, which is available to authorized users.

## Introduction

*N*-methyl-d-aspartate (NMDA) receptor hypofunction has been proposed to contribute to the symptoms of schizophrenia. Acute administration of NMDA receptor antagonists produces transient psychosis-like activity in healthy humans and exacerbates the pre-existing symptoms in stabilised schizophrenic individuals (Krystal et al. 1994; Lahti et al. 1995). This suggests that NMDA receptor antagonists can interact with neuronal networks that mediate psychosis.

Oscillations recorded in local field potentials (LFP) or electroencephalograms (EEG) are generated by synchronous changes in the membrane potentials of a population of neurons which can be a few hundred thousand for LFP and several million for EEG recordings. Abnormal oscillatory activity recorded in EEG has been associated with schizophrenia (Uhlhaas and Singer 2010; Spencer 2011). An increasing number of studies have used these techniques in attempts to understand how NMDA receptor hypofunction influences regional brain activity. In experimental rodents, acute application of NMDA receptor antagonists is known to influence oscillations recorded in LFP (Ma and Leung 2000; Middleton et al. 2008; Roopun et al. 2008; Kocsis 2012) and electrocorticograms (Pinault 2008; Phillips et al. 2012).

The nucleus accumbens (NAc) is a brain region that has been implicated widely in the pathophysiology of schizophrenia (Grace 2000). Previously, we identified a high-frequency oscillation (HFO; 130–180 Hz) recorded in the rat NAc that is enhanced substantially following systemic or local administration of NMDA receptor antagonists (Hunt et al. 2006). In the NAc, increases in the power of HFO produced by NMDA receptor antagonists predominate well above changes found in other frequency bands (Hunt et al. 2010). In recent years, a number of studies have demonstrated that a HFO band can be recorded in a variety of neuroanatomical and functionally distinct regions following injection of NMDA receptor antagonist (Hunt et al. 2011; Nicolas et al. 2011; Kulikova et al. 2012; Phillips et al. 2012; Ji et al. 2013; Hiyoshi et al. 2014). Tetrodotoxin infusion to the NAc produces profound reductions in HFO power which correlate with changes recorded in distant cortical sites, indicating the NAc may be an important generator of HFO (Olszewski et al. 2013a). In rats, atypical antipsychotic drugs (i.e. clozapine) reduce the frequency of accumbal HFO to a greater extent than typical drugs (i.e. haloperidol) (Olszewski et al. 2013b). Whilst both drug types bind to dopamine D2 receptors, atypical drugs differ from typical drugs in their mechanism of action. The improved efficacy of atypical antipsychotic compounds has been attributed to their additional powerful action at serotonergic receptors, perhaps most notably 5-HT_2A_, but also 5-HT_1A_ and 5-HT_7_ receptors (Meltzer 1999). Compounds that bind to serotonergic receptor subtypes can block NMDA receptor antagonist-induced hyperlocomotion (Gleason and Shannon 1997) and attentional impairments (Higgins et al. 2003; Mirjana et al. 2004) and may reverse dysfunctional glutamatergic transmission produced by NMDA receptor antagonists (Ceglia et al. 2004; McOmish et al. 2012). Amongst the array of actions at other receptor systems, atypical antipsychotics are known to antagonise histamine H_3_ receptors (Rodrigues et al. 1995) and potentiate NMDA receptor currents (Wittmann et al. 2005). These actions may also contribute to the clinical profile of drugs such as clozapine. Experimental compounds that interact at these sites have been proposed as novel antipsychotics or adjunctive therapy in the treatment of schizophrenia (Wittmann et al. 2005; Ito 2009).

To date, the majority of animal model studies have focused on rats, mainly due to the convenience of the implantation in this species and for comparison with earlier studies. Effects of NMDA receptor antagonists on LFP oscillations recorded in mice, which are also a widely used experimental animal, are less well investigated. In freely moving mice, ketamine has been shown to decrease the power of basal hippocampal theta (3–12 Hz) and increase gamma (30–80 Hz) power (Lazarewicz et al. 2010); however, effects on HFO remain largely unknown. A recent study demonstrated that administration of ketamine generated significantly higher cortical power in the HFO band in Sp4 hypomorphic mice (decreased expression of NR1 protein) compared with wild-type siblings (Ji et al. 2013). However, in this study, a negligible increase in HFO power was observed in wild-type mice despite the relatively high dose of ketamine used (50 mg/kg). This may suggest a difference in the generation of HFO between species. Since, in rats, the NAc appears to be a locus for generation of HFO, this prompted us to investigate whether the same holds true for mice. This issue gains importance considering the increasing number of genetic mouse models and emergence of optogenetic techniques reliant on modified genetic background for neuronal specificity. In this study, we also used a pharmacological strategy to shed light on the mechanism through which atypical antipsychotics reduce the frequency of HFO.

## Methods

### Surgery

Thirty-four male C57BL/6 and 10 BALB/c mice (20–28 g) were implanted with a pair of twisted stainless steel electrodes (125 μm, Science Products, Germany) in the NAc (AP, 1.3 mm; ML, 0.75 mm; DV, 4.2 mm). In all cases, a silver wire was used as ground/reference electrode connected to a screw posterior to the bregma. Mice were housed individually with access to water and food ad libitum. The location of tips of electrode (electrolytic lesion) was determined on 40-μm Cresyl violet-stained sections.

### Experimental groups

Mice were placed in a recording chamber (35 × 35 × 42 cm). LFP were recorded through a JFET pre-amplifier. The signal was relayed through a commutator (Plastics One) amplified ×1000, filtered 0.1–1 kHz (A-M Systems, USA) and digitised 4 kHz (Micro1401, CED, Cambridge, UK). Data were stored on a PC for offline analysis.

Mice were assigned to six experimental groups (see Supplementary Table 1). *Group 1* (*n* = 4) includes 20 min baseline followed by injection of 10, 25 and 50 mg/kg ketamine or vehicle; *group 2* (*n* = 5) includes 20 min baseline followed by injection of 0.05, 0.1, 0.25 and 0.5 mg/kg MK801 or vehicle; *group 3* (*n* = 6) includes 20 min baseline followed by injection of 0.25 mg/kg MK801, followed 30 min later by injection of 1, 5 and 15 mg/kg clozapine or vehicle; group *4* (*n* = 7) includes 20 min baseline followed by 0.25 mg/kg MK801, followed 30 min later by 2.0 g/kg glycine, 75 mg/kg NMDA or vehicle; and *group 5* (*n* = 6) includes 20 min baseline followed by injection of 0.25 mg/kg MK801, followed 30 min later by 1.0 mg/kg 8-OH-DPAT, 1.0 mg/kg MDL 11, 939 or vehicle. Some mice (*n* = 10) from groups 4 and 5 also received injection (intraperitoneal (i.p.)) of 1.5 mg/kg haloperidol 30 min post-MK801, and locomotor activity (LMA) was evaluated in four of these mice. *Group 6* (*n* = 5) includes 20 min baseline followed by 0.25 mg/kg MK801, followed 30 min later by injection of a low dose of haloperidol (0.15 mg/kg) or vehicle and *group 7* (*n* = 6) includes 20 min baseline followed 20 min later by injection of 0.25 mg/kg MK801, followed 30 min later by injection of 1.0 mg/kg SB269970, 5.0 mg/kg BF2649 or vehicle. With exception of group 2, locomotor activity was assessed by beam breaks (Columbus Instruments, USA). The experiments were performed according to Latin-square design, whereby each animal with the individual groups received all injection(s) assigned in a pseudorandomised order. All experiments were conducted in accordance with the European Community guidelines on the Care and Use of Laboratory Animals (86/609/EEC) and approved by a local ethics committee.

Selection of drugs and doses were based on published findings demonstrating reversal of the effects of NMDA receptor antagonists on motor, cognitive or neurotransmitter effects (Gleason and Shannon 1997; Ninan and Kulkarni 1998; Higgins et al. 2003; Mirjana et al. 2004; Ligneau et al. 2007; Horiguchi et al. 2011; Brabant et al. 2013; Nikiforuk et al. 2013).

### Data analysis

LFP signals were inspected for movement artefacts which when present were removed. These were infrequent events which typically corresponded to large amplitude deflections of the raw LFP, usually associated with impact of the headset with the wall of the recording chamber. Mean power spectra of the raw LFP were carried out on successive 60-s data blocks using a fast Fourier transform of 4096 points (Spike 2). Total power (130–180 Hz) and dominant frequency were calculated. We also analysed 10-min means at the end of the 60-min time course, unless stated otherwise.

### Statistics

Data were analysed using analysis of variance (ANOVA) with time as the repeated measure followed by the Bonferroni post hoc test. Means of 10 min with or without drug were compared using one-way ANOVA followed by the Bonferroni post hoc test or Student’s paired *t* tests if only two groups were compared. Differences were considered significant when *p* < 0.05.

## Results

### NMDA receptor antagonists dose-dependently increase the power and frequency of HFO in the mouse nucleus accumbens

**Fig. 1:**
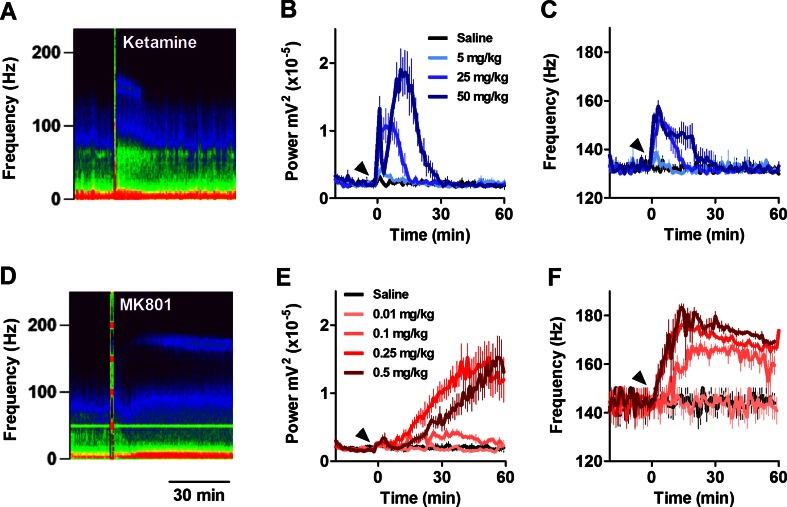
NMDA receptor antagonists dose-dependently increase the power and frequency of HFO in the mouse NAc. **a** Accumbal LFP expressed as spectrograms showing the effect of intraperitoneal injection of 25 mg/kg ketamine. **b**, **c** Time courses showing the effect of different doses of ketamine (5, 25, 50 mg/kg, *N* = 4) on the power and frequency of HFO. **d** Accumbal LFP expressed as spectrograms showing the effect of intraperitoneal injection of 0.25 mg/kg MK801. **e**, **f** Time courses showing the effect of different doses of MK801 (0.01, 0.1, 0.25, 0.5 mg/kg, *N* = 5) on the power and frequency of HFO. Saline was used as vehicle in both instances. *Arrows* indicate time of injection. Values are mean ± SEM

Power spectra analysis of LFP recorded in the mouse NAc revealed the occurrence of weak spontaneous HFO (130–180 Hz) in some, but not all mice. When present, spontaneous HFO were visible, typically as a small bump in the power spectra around 140 Hz. LFP were recorded before and up to 1 h post-i.p. injection of ketamine (10, 25, 50 mg/kg), MK801 (0.01, 0.1, 0.25, 0.5 mg/kg) or vehicle. Representative time courses expressed as spectrograms for each drug are shown in Fig. 1a, d. Repeated-measure ANOVA for HFO power revealed a significant group × time interaction (*F*(222, 888) = 12.68; *p* < 0.0001). Bonferroni post hoc analysis revealed HFO power significantly (*p* < 0.001) increased between 1 and 10 min post-injection of 25 mg/kg ketamine. The highest dose (50 mg/kg) produced a biphasic increase in HFO power, characterised by an immediate (phase 1) increase occurring in the first minute (*p* < 0.001), followed by a return to baseline power and a second increase (phase 2) in power (*p* < 0.01) occurring 5 to 20 min post-injection. There were no significant differences in power between saline and the 10-mg/kg dose of ketamine. Consistent with our findings from rats, the power of HFO positively correlated with beam breaks (Supplementary Fig. 1). Repeated-measure ANOVA for HFO also revealed a group × time interaction (*F*(222, 888) = 4.73; *p* < 0.0001), with Bonferroni post hoc analysis revealing that 25 mg/kg ketamine increased HFO between 1 and 10 min (*p* < 0.05), and 50 mg/kg ketamine associated with increases between 1 and 19 min post-injection (*p* < 0.001).

Systemic injection of MK801 also dose-dependently increased the power and frequency of HFO (Fig. 1e, f). Analyses of the time courses revealed significant group × time interactions for both HFO power and frequency (*F*(308, 1540) ≤ 10.59; *p* < 0.001). Increases in HFO power were found for 0.25 and 0.5 mg/kg MK801, vs. saline, from 19 to 29 min, respectively. Increases in frequency, with respect to saline, occurred from 19 min (0.1 mg/kg) to 8 min (0.25 and 0.5 mg/kg).

### Clozapine dose-dependently reduces MK801-enhanced HFO in the mouse nucleus accumbens

**Fig. 2:**
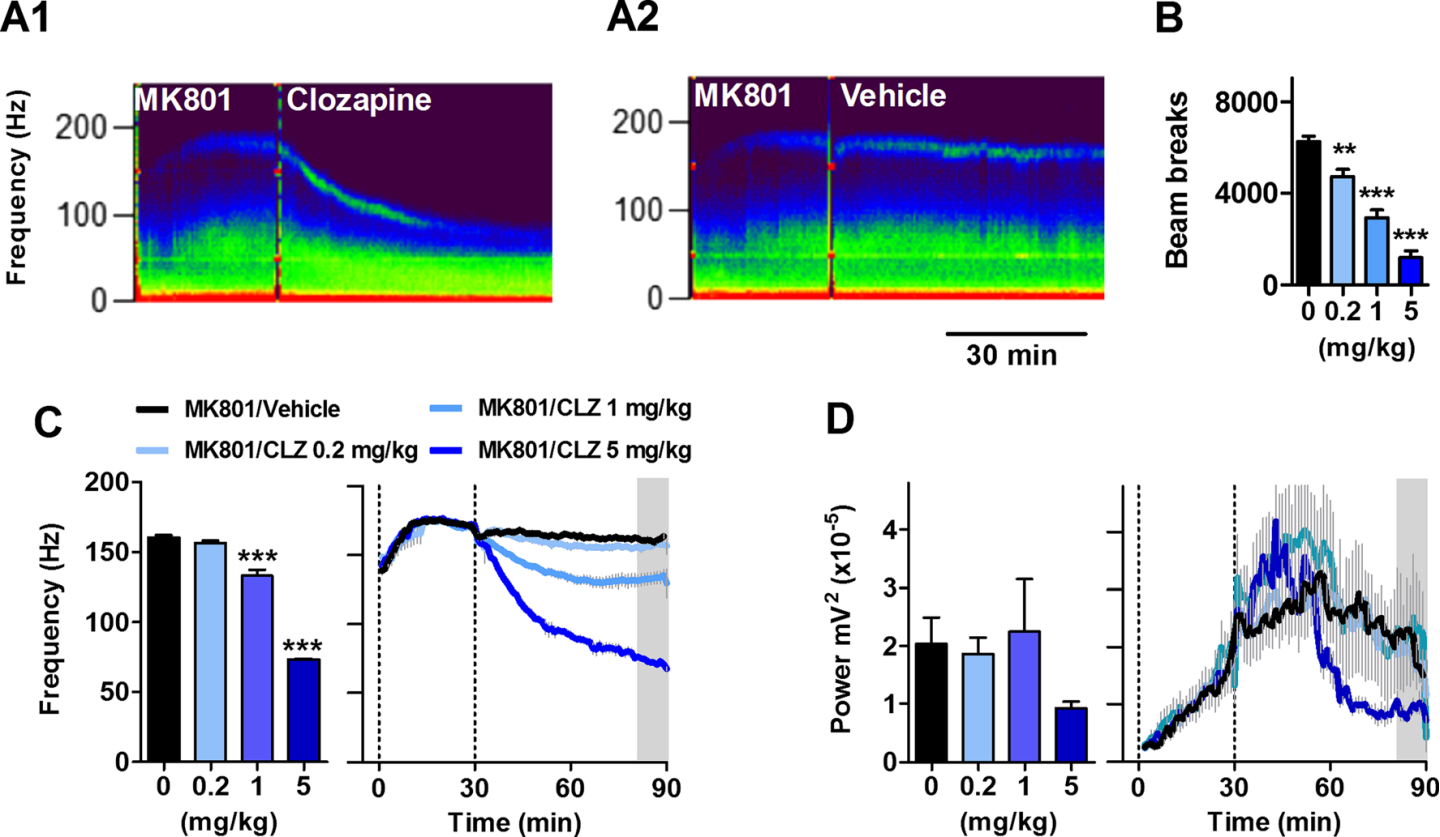
The atypical antipsychotic clozapine dose-dependently decreases the frequency of HFO in the mouse NAc. *A1*, *A2* Example spectrograms taken from the same mouse showing the effect of intraperitoneal injection of clozapine or vehicle on 0.25 mg/kg MK801-enhanced HFO. *B* Total number of beam breaks after injection of clozapine. *C*, *D* Histograms showing the effect of different doses of clozapine (0.2, 1, 5 mg/kg, *N* = 6) or vehicle on the frequency and power of MK801-enhanced HFO. Values are mean ± SEM for a 10-min period (approximately 50-60 min) post-injection of clozapine and indicated by the *shaded area* shown on the time courses in the *right-hand figure*. **p < 0.01; ***p < 0.001 compared with vehicle

We next examined the effect of i.p. injection of the prototypical atypical antipsychotic compound, clozapine on HFO 30 min post-injection of 0.25 mg/kg MK801. Representative spectrograms showing the effect of clozapine or vehicle on HFO from the same mouse are shown in Fig. 2 (A1, A2). Clozapine dose-dependently reduced motor activation induced by injection of MK801 (one-way ANOVA, *F*(3, 5) = 64.47; *p* < 0.0001; Fig. 2b). Bonferroni post hoc analysis showed differences between vehicle and all doses of clozapine (*p* < 0.01). There were also differences between the lowest dose of clozapine and the medium and higher doses (*p* < 0.01) as well as between the medium and higher doses (*p* < 0.01).

Clozapine (0.2, 1, 5 mg/kg) dose-dependently reduced the frequency of MK801-enhanced HFO (*F*(3, 23) = 250.6; *p* < 0.0001, repeated-measure one-way ANOVA; Fig. 2c). Bonferroni post hoc test revealed significant differences between vehicle vs. 1 and 5 mg/kg doses of clozapine (*p* < 0.001). Differences were also found between the lowest dose at 0.2 mg/kg and higher doses and between the 1- and 5-mg/kg doses (*p* < 0.001). We did not find a significant effect of clozapine on MK801-induced HFO power (*F*(3, 23) = 1.6; *p* = 0.23, repeated-measure one-way ANOVA). Complete time courses are shown in Fig. 2d. Repeated-measure ANOVA of the time course revealed a significant group × time interaction (*F*(243, 1620) = 54.1; *p* < 0.0001). Bonferroni post hoc revealed the highest dose (5 mg/kg), produced a rapid effect on HFO with significant differences found from 5 min (*p* < 0.01) post-injection. The middle dose (1 mg/kg) produced significant reductions in frequency from 10 min post-injection (*p* < 0.01) with respect to vehicle. No differences were found between the lowest dose of clozapine and vehicle.

### Haloperidol does not influence the frequency of HFO in the mouse nucleus accumbens

**Fig. 3:**
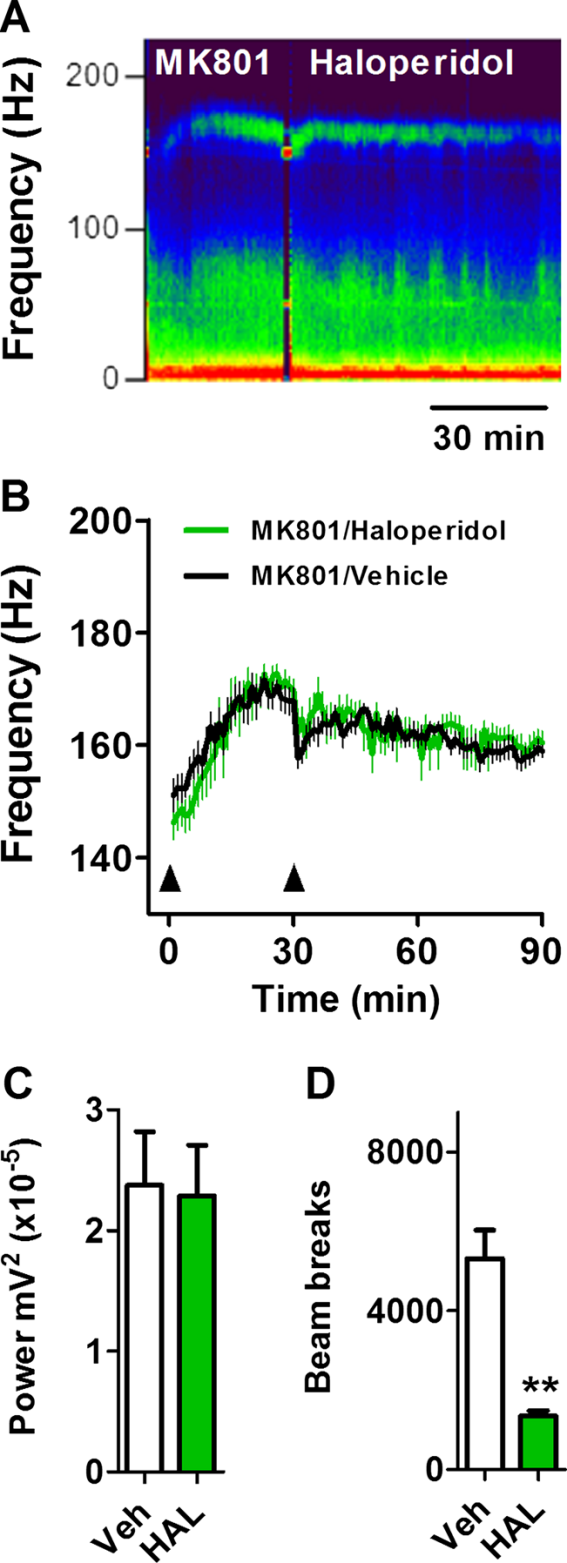
The typical antipsychotic haloperidol does not reduce the frequency or power of HFO in the mouse NAc. **a** Example spectrogram from a representative mouse showing the effect of 1.5 mg/kg i.p. haloperidol on 0.25 mg/kg MK801-enhanced HFO. **b** Time courses showing the frequency of MK801-enhanced HFO before and after injection of haloperidol or vehicle (*N* = 10). *Arrows* indicate injection of MK801and haloperidol, respectively. **c** Average power of MK801-enhanced HFO after injection of haloperidol or vehicle. **d** Total number of beam breaks after injection of haloperidol was evaluated in four of the mice. Values are mean ± SEM. **p < 0.01 compared with vehicle

The effect of i.p. injection of the typical antipsychotic, haloperidol, on MK801-enhanced HFO is shown by a spectrogram in Fig. 3a. Injection of haloperidol (1.5 mg/kg) did not significantly affect the frequency (group × time, *F*(83, 1512) = 0.69; *p* > 0.05; Fig. 3b) or power (*t* = 0.172; *df* = 9; *p* = 0.867; Fig. 3c). To test whether the administered dose had any effect on the animals, we also measured locomotor activity. Although this dose of haloperidol did not influence HFO power or frequency, it did reduce the amount of locomotor activation produced by MK801 (*t* = 6.56; *df* = 3; *p* = 0.0072; Fig. 3d). We also examined the effect of a lower dose of haloperidol (0.15 mg/kg) in a separate group of mice. The time course is shown in Supplementary Fig. 2. The lower dose did not significantly influence the frequency (group × time, *F*(85, 680) = 0.65; *p* > 0.05) or power (group × time, *F*(85, 680) = 0.59; *p* > 0.05) of MK801-enhanced HFO.

### Glycine and NMDA reduce MK801-enhanced HFO

**Fig. 4:**
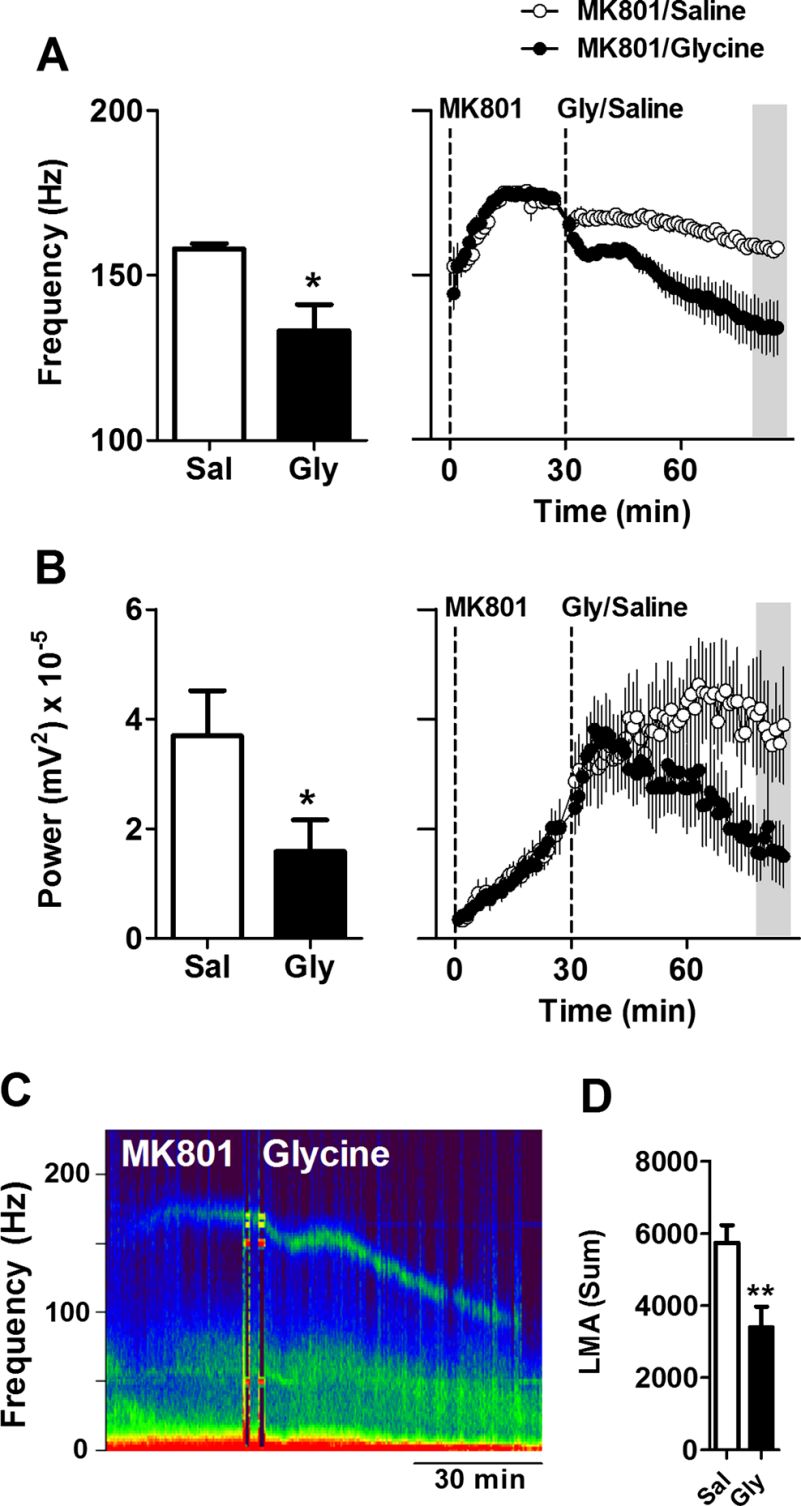
Glycine reduces the frequency and power of MK801-enhanced HFO in mice. **a**, **b** Histograms showing the effect of 2 g/kg glycine or saline on the frequency and power of MK801-enhanced HFO. Values are mean ± SEM for a 10-min period (approximately 50–60 min) post-injection of glycine and indicated by the *shaded area* shown on the time courses in the *right-hand figure* (*N* = 7). **c** LFP expressed as a spectrogram from a representative mouse. **d** Total number of beam breaks post-injection of glycine or saline. Glycine reduces MK801-induced hyperlocomotion. *p < 0.05; **p < 0.01 compared with vehicle

Glycine is a co-agonist of NMDA receptors which at high doses has been found to have antipsychotic properties in humans (Balu and Coyle 2015). We examined the effect of glycine at 2 g/kg approximately 30 min after injection of MK801. Glycine produced a long-lasting reduction in the frequency of HFO. At 1 h post-treatment of glycine, the frequency of HFO was 133.3 ± 7.9 Hz compared with 158.1 ± 1.5 Hz for mice receiving saline (*p* = 0.028, paired *t* test; Fig. 4a). Analysis of the time course with repeated-measure ANOVA revealed a group × time interaction (*F*(81, 972) = 6.99; *p* < 0.0001). Significant reductions in HFO (*p* < 0.05) occurred from 15 min post-injection of glycine. Interestingly, the reduction in frequency appeared to occur in two phases, an initial rapid drop in frequency by approximately 15 Hz, followed by a more gradual reduction in frequency over the course of the experiment. The power of HFO shows that 1 h post-glycine was also significantly lower compared with saline controls (*p* = 0.0125, paired *t* test; Fig. 4b). A representative spectrogram showing the effect of glycine on MK801-enhanced HFO is shown in Fig. 4c. Consistent with the findings of others (Nilsson et al. 1997), glycine also reduced MK801-enhanced locomotion with respect to saline (*p* = 0.026, paired *t* test; Fig. 4d).

**Fig. 5:**
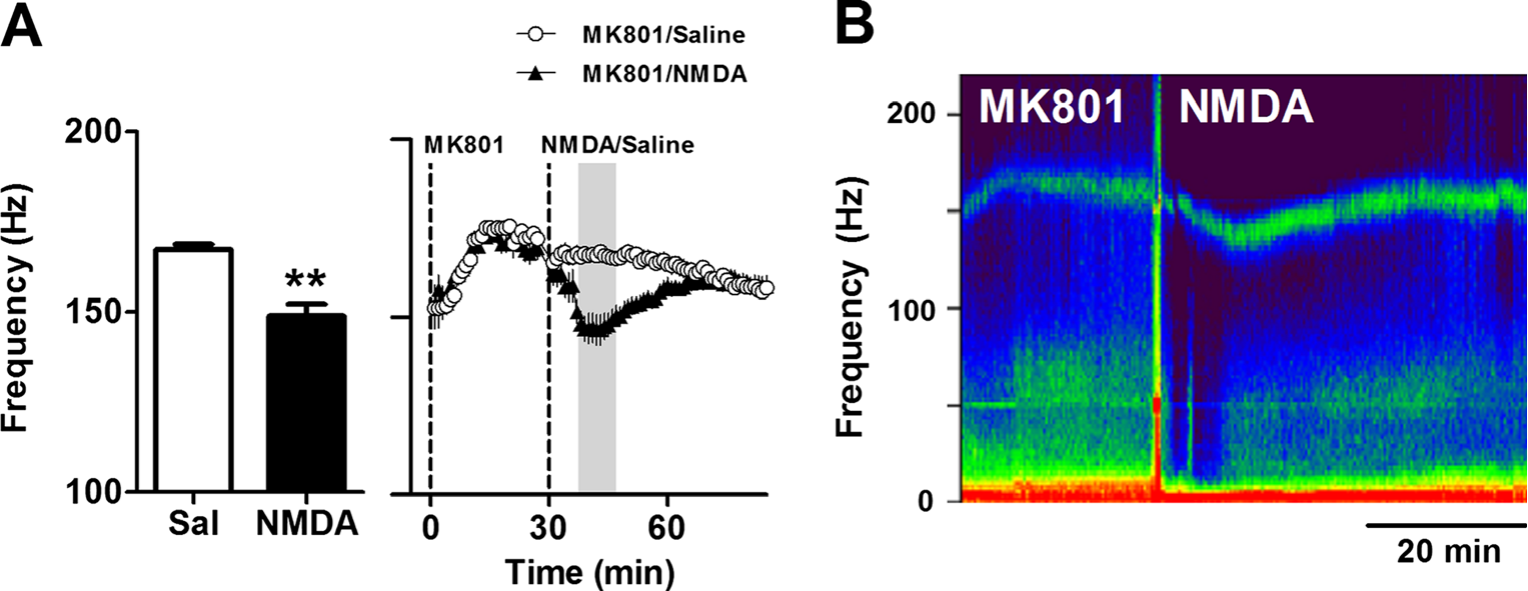
NMDA reduces the frequency of MK801-enhanced HFO in mice. **a** Histogram showing the effect of 70 mg/kg NMDA or saline on the frequency of MK801-enhanced HFO (*N* = 7). Values are mean ± SEM for a 10-min period approximately 10–20 min post-injection of NMDA and indicated by the *shaded area* shown on the time courses in the *right-hand figure*. This experiment was conducted using mice from the glycine study; therefore, control values shown are those from Fig. 4. **b** LFP expressed as a spectrogram from a representative mouse. **p < 0.01 compared with vehicle

Since glycine is known to potentiate NMDA receptor function, we next examined the effect of intraperitoneal injection of 75 mg/kg NMDA on the frequency of MK801-enhanced HFO. We found that NMDA produced an immediate and short-lasting reduction in frequency which typically lasted around 30 min but did not influence the power of HFO. Mean frequency at 10–20 min post-injection of 75 mg/kg NMDA was 149 ± 3.2 Hz vs. 167.3 ± 1.4 Hz from saline-injected mice (*p* = 0.0014, paired *t* test; Fig. 5a). Analysis of the time course, using repeated-measure ANOVA, revealed a group × time interaction (*F*(81, 972) = 9.63; *p* < 0.0001). Significant reductions in HFO frequency (*p* < 0.01) occurred between 7 and 22 min post-injection of NMDA. Time courses are also shown in Fig. 5a, and a representative spectrogram is shown in Fig. 5b.

### Stimulation of 5-HT_1A_ but not blockade of 5-HT_2A_, 5-HT_7_ or H_3_ receptors reduced the frequency of MK801-enhanced HFO

**Fig. 6:**
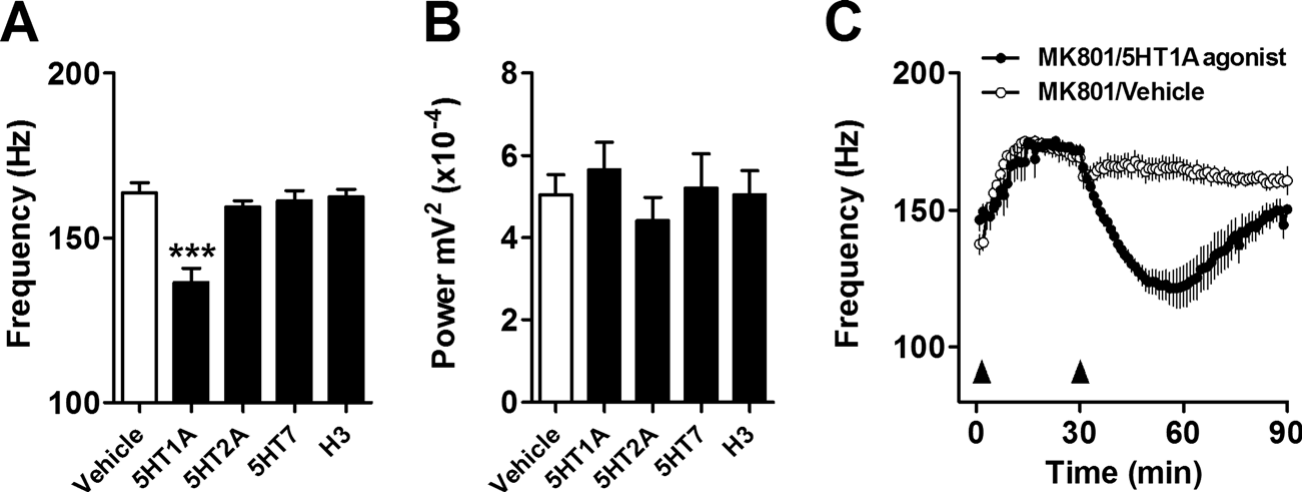
Stimulation of 5-HT_1A_ receptors produces a short-lasting reduction in HFO. **a**, **b** Frequency and power of MK801-enhanced HFO (average 60 min) post-injection of 1.0 mg/kg 8-OH-DPAT (5-HT_1A_ agonist), 1.0 mg/kg MDL 11939 (5-HT_2A_ antagonist), 1.0 mg/kg SB269970 (5-HT_7_ antagonist) and 5.0 mg/kg BF2649 (H_3_ inverse agonist). These compounds mimic some of the different pharmacological activities of clozapine at specific receptor targets (*N* = 5–6/group). Values are mean ± SEM. **c** Time course showing the effect of intraperitoneal injection of the potent 5-HT_1A_ agonist 8-OH-DPAT on the frequency of MK801-enhanced HFO. *First arrow* indicates injection of 0.25 mg/kg MK801; *second arrow* indicates injection of 8-OH-DPAT or vehicle. ***p < 0.001

One-way ANOVA revealed a significant effect on the frequency of MK801-enhanced HFO (*F*(4, 29) = 14.33; *p* < 0.0001; Fig. 6a). Bonferroni post hoc test revealed a significant reduction after injection of 8-OH-DPAT, a potent 5-HT_1A_ agonist, compared with vehicle and the other drugs tested (*p* < 0.001). There were no significant changes in HFO frequency after systemic injection of antagonists at 5-HT_2A_ (1 mg/kg MDL11939) and 5-HT_7_ receptors (1 mg/kg SB269970) or an H_3_ inverse agonist (5 mg/kg BF2649) with respect to control. No drug influenced the power of MK801-enhanced HFO (*F*(4, 29) = 0.74; *p* = 0.75; Fig. 6b). Time course showing the effect of 8-OH-DPAT on the frequency of MK801-enhanced HFO is shown in Fig. 6c. Analysis of the time course, using repeated-measure ANOVA, revealed a group × time interaction (*F*(85, 850) = 10.4; *p* < 0.0001). Significant reductions in HFO (*p* < 0.01) occurred between 9 and 46 min post-injection of 8-OH-DPAT.

### HFO are smaller amplitude but faster frequency in mice compared with rats

We carried out a further experiment to compare spontaneous and enhanced HFO in the BALB/c (*n* = 10) compared with the C57BL/6 strain. Due to the relatively small power of HFO at baseline, and the lack of a discernible peak in the spectra, it was not possible to consistently evaluate its frequency at baseline. We did, however, evaluate the integrated power for the HFO band (130–180 Hz) and found no significant difference for HFO power at baseline (*t* = 1.2; *df* = 35; *p* = 0.23) or post-injection of 0.25 mg/kg MK801 (*t* = 1.5; *df* = 35; *p* = 0.13). However, the frequency of MK801-enhanced HFO was significantly higher in C57BL/6 compared with BALB/c (*t* = 3.1; *df* = 35; *p* = 0.0034).

We conducted further analyses to include data from our previously published rat studies to compare HFO in C57BL/6, BALB/c mice and Wistar rats. Analysis of integrated HFO power at baseline revealed significantly smaller (*p* < 0.01) power in both strains of mice compared with rats (one-way ANOVA, *F*(2, 66) = 9.8; *p* < 0.0002). The power between BALB/c and C57BL/6 was not significantly different.

We also examined the effect of MK801-enhanced HFO using data from our previously published rat studies at a dose of 0.15 mg/kg. Although the dose of MK801 was lower in rats, analysis of the total HFO power 30 min post-injection revealed that the power of HFO was significantly higher in rats (*p* < 0.01) compared with both strains of mice, and no difference between the mouse strains (one-way ANOVA, *F*(2, 66) = 29.9; *p* < 0.0001). The frequency of MK801-enhanced HFO was also significantly (*p* < 0.001) higher in mice (C57BL/6, 170.1 ± 1.2 Hz; BALB/c, 163.2 ± 1.3 Hz) compared with rats (Wistar, 143.9 ± 1.2 Hz; one-way ANOVA, *F*(2, 64) = 110.3; *p* < 0.0001). We also found that the frequency of HFO post-MK801 was significantly higher in C57BL/6 compared with BALB/c mice (*p* < 0.05).

Clozapine-induced reduction in HFO frequency was more substantial in C57BL/6 mice compared with Wistar rats (*p* < 0.001). In mice, clozapine (5 mg/kg) reduced the frequency by almost 100 Hz, whilst in rats a higher dose of 15 mg/kg reduced HFO by around 50 Hz. A dose of clozapine at 5 mg/kg in Wistar rats produced small effects on HFO (Olszewski et al. 2013b).

## Discussion

NMDA receptor antagonists produced a sustained increase in the power and frequency of HFO in the mouse NAc. In the presence of MK801, the atypical antipsychotic drug, clozapine, dose-dependently reduced the frequency of HFO, whilst the typical antipsychotic drug, haloperidol, was without effect. Although we did observe some species differences between mice and rats, the findings reported here are broadly in line with our previous studies using rats (Hunt et al. 2006; Olszewski et al. 2013b).

### Clozapine and glycine reduce HFO frequency

Clozapine dose-dependently reduced the frequency of HFO in mice; at the highest dose, an approximate 80 Hz reduction was observed. Effects of clozapine were examined against a background of MK801-enhanced HFO. The dose of MK801 we used is similar to earlier studies where behavioural effects of antipsychotic compounds have been evaluated (Ninan and Kulkarni 1998; Bradford et al. 2010). As expected, clozapine dose-dependently reduced MK801-induced locomotor activity. Haloperidol, at a dose that produced an equivalent reduction in locomotor activity, did not influence the frequency of power of HFO, in line with our findings in rats (Olszewski et al. 2013b). A low dose of haloperidol (0.15 mg/kg), close to the concentration reported to reverse effect of phencyclidine-induced increase in cortical pyramidal cell firing (Kargieman et al. 2007), was also without effect on the increases in HFO power or frequency produced by MK801. These findings corroborate our earlier study in rats which indicate that reduction in HFO may be an electrophysiological signature of ‘atypicality’. Since the baseline power of HFO, in mice, was often negligible, we could not adequately determine the effect of clozapine on baseline activity. However, in our previous study, using rats—which typically display a clear peak in the HFO range, we observed clozapine, but not haloperidol, reduced the frequency of HFO, indicating that atypical drugs differentially interact with the fundamental mechanisms responsible for the generation of HFO in the NAc.

Enhancing NMDA receptor neurotransmission at the glycine modulatory site has been proposed as an effective therapeutic strategy for the treatment of schizophrenia (Balu and Coyle 2015). There is good evidence from pre-clinical studies that demonstrates glycine and compounds which enhance synaptic glycine levels possess antipsychotic efficacy. In animal models, these compounds reduce psychotomimetic-induced hyperlocomotion (Boulay et al. 2008), as well as cognitive deficits induced by NMDA receptor antagonists, including attentional deficits, novel object recognition and latent inhibition (Depoortere et al. 2005; Karasawa et al. 2008; Black et al. 2009). Glycine has been shown to reverse deficits in pre-pulse inhibition in a rat neonatal ventral hippocampal lesion model (Le Pen et al. 2003). In this study, we found that glycine also produced a robust reduction in the frequency of HFO. This is potentially interesting since it corroborates our previous finding that drugs with antipsychotic properties, particularly those effective in controlling the negative and cognitive symptoms of schizophrenia, reduce the frequency of HFO in the NAc (Olszewski et al. 2013b).

### Modulation of accumbal HFO by NMDA and 5-HT_1A_ receptors

Potentiation of NMDA receptor function either directly (injection of NMDA) or indirectly (glycine) reduced the frequency of HFO. NMDA receptors play an important role in the activity of medium spiny neurons, in particular the transition from hyperpolarised ‘down’ states to ‘up’ ready-to-fire states (Vergara et al. 2003; Wolf et al. 2005). Both clozapine and risperidone potentiate NMDA receptor neurotransmission in cortical neurons (Konradsson et al. 2006). Additionally, using whole patch cell recordings, it has been shown that bath application of clozapine, but not haloperidol or the 5-HT_2A_ antagonist MDL100907, potentiates NMDA receptor currents in NAc neurons (Wittmann et al. 2005). Notably, sulpiride, which we showed previously can reduce HFO in rats, also significantly increased NMDA receptor currents in NAc neurons (Wittmann et al. 2005). These findings point to a pivotal role played by NMDA receptors in influencing the frequency of HFO recorded in the NAc. However, other receptor systems can also influence the frequency of HFO. We found that 8-OH-DPAT, an agonist at 5-HT_1A_ receptors, reduced the frequency of NMDA receptor antagonist-enhanced HFO. Clozapine and several other atypical antipsychotic drugs possess partial agonistic activity at 5-HT_1A_ receptors (for review, see Celada et al. 2013). Compounds which possess combined 5-HT_2A_/D_2_ properties with direct 5-HT_1A_ receptor agonistic properties are active in a broad range of models of schizophrenia. These observations have led to the development of novel antipsychotic compounds such as aripiprazole and lurasidone (for review, see Newman-Tancredi and Kleven 2011). The present study shows that NMDA and 5-HT_1A_ receptors can influence the frequency of accumbal HFO, whilst it may be inferred we have not categorically demonstrated that clozapine exerts its effects on HFO through either of these receptors. Clozapine possesses antagonist properties at many other receptors, including 5-HT_2A_ (Canton et al. 1994), 5-HT_7_ (Roth et al. 1994) and H_3_ receptors (Rodrigues et al. 1995). Blockade of these receptors did not influence the frequency of MK801-enhanced HFO, indicating that clozapine and other antipsychotic compounds do not exert their effects on HFO through these receptors.

NMDA receptor antagonists appear to preferentially block NMDA receptors in cortical interneurons (Homayoun and Moghaddam 2007) and GABAergic neurons of the reticular nucleus of the thalamus (Troyano-Rodriguez et al. 2014). Although the major cell type in the NAc is medium spiny projection neurons, which comprise between 90 and 95 %, several different classes of interneurons are known to be present, including calbindin, parvalbumin, somatostatin and large cholinergic subtypes (Berke 2011; Russo and Nestler 2013). Whether NMDA receptor antagonists preferentially target specific neuronal subtypes warrants investigation, possibly using optogenetic techniques. As well as local mechanisms, afferent projections are likely to play a role in the generation of HFO. Indeed, we have shown previously that local injection of clozapine to the NAc of rats does not mimic the effects observed after systemic injection (Olszewski et al. 2013b). The NAc receives glutamatergic projections from the prefrontal cortex, amygdala, hippocampus and thalamus (Groenewegen et al. 1999), and a dense dopaminergic projection from the ventral tegmental area. Although NMDA receptor antagonists can increase the firing of prefrontal cortical neurons (Homayoun and Moghaddam 2007), infusion of TTX to the prelimbic region of the prefrontal cortex did not disrupt MK801-enhanced HFO in rats (Olszewski et al. 2013a), suggesting that this region does not drive HFO in the NAc.

### Accumbal HFO in mice and rats

In mice, spontaneous HFO were rarely observed, and when present, they could only be detected as a very small bump in the averaged power spectra. This is quite different in rats where spontaneous HFO are clearly visible in the majority of recordings from the NAc. Given the smaller power at baseline in mice, it is not surprising that HFO power post-NMDA receptor antagonist injection was also smaller. However, the fundamental effects produced by NMDA receptor antagonists on mechanisms generating HFO appear to be similar in mice and rats. For example, the temporal changes seen in HFO power generated after NMDA receptor antagonists closely follow those previously reported in rats (Hunt et al. 2006; Nicolas et al. 2011). In both mice and rats, ketamine produced a rapid short-lasting increase in power, whilst MK801 generated HFO which persisted throughout the course of the experiments. Changes in locomotion and HFO power positively correlated a finding consistent with our previous study (Hunt et al. 2006).

With respect to HFO, the frequency of post-injection of MK801 was 170.1 ± 1.2 Hz compared with 146.3 ± 1.2 Hz in rats. Mice strains vary substantially in their behavioural response to NMDA receptor antagonists and antipsychotic drugs. Therefore, we also examined HFO in the behaviourally distinct BALB/c strain of mice (Holmes et al. 2002; Sik et al. 2003; Bothe et al. 2005). Like C57BL/6 mice, HFO in the BALB/c strain was lower in power but higher in frequency compared with Wistar rats. Although HFO power was not different between the two mice strains, we found that the frequency of HFO was slightly lower in BALB/c mice.

When we examined the effects produced by clozapine, there were certain notable differences between our mice and previously published rat study. Firstly, the dose of clozapine efficacious at reducing HFO was lower in mice than rats; a dose of 5 mg/kg robustly reduced the frequency, by around 80 Hz, in mice, whilst in rats, the same dose produced only weak 10–20 Hz reductions. Additionally, at this dose in mice after a subtle increase in power, we observed a dramatic reduction in power which typically occurred when HFO reached approximately 100 Hz. In rats, clozapine potentiated the power of HFO whilst simultaneously reducing HFO. One possible explanation for this difference is methodology. In our previous rat study, the dose of MK801 used was submaximal (Olszewski et al. 2013b). In the current study, using mice, we used a dose of MK801 that produced close to maximal increases in HFO power; therefore, further potentiation of HFO power may not be physiologically possible. Notwithstanding, although there are certain characteristic differences between the species, the fundamental effects produced by NMDA receptor antagonists and antipsychotics on HFO appear broadly similar in mice and rats.

## Conclusions

These findings show that although HFO power and frequency are different in rats and mice, the effects produced by psychotomimetic and antipsychotic compounds are broadly similar in both species. These data also suggest that atypical antipsychotic drugs may reduce the frequency of HFO by interacting with NMDA and/or 5-HT_1A_ receptors.

## Electronic supplementary material

Supplementary Fig. 1 Correlation between changes in ketamine-induced locomotion and HFO power (GIF 76 kb)

High-resolution image (TIFF 447 kb)

Supplementary Fig. 2 Frequency and power of MK801-enhanced HFO after injection of 0.15 mg/kg haloperidol or vehicle (GIF 33 kb)

High-resolution image (TIFF 238 kb)

Supplementary Table 1 Summary of the experimental groups used in the study (DOC 27 kb)
