# Supplementary figures and images for: Effects of NMDA receptor antagonists and antipsychotics on high frequency oscillations recorded in the nucleus accumbens of freely moving mice
Source: Psychopharmacology (Berl). 2015 Oct 8;232(24):4525–35. doi: 10.1007/s00213-015-4073-0 (PMC4646921; doi:10.1007/s00213-015-4073-0)

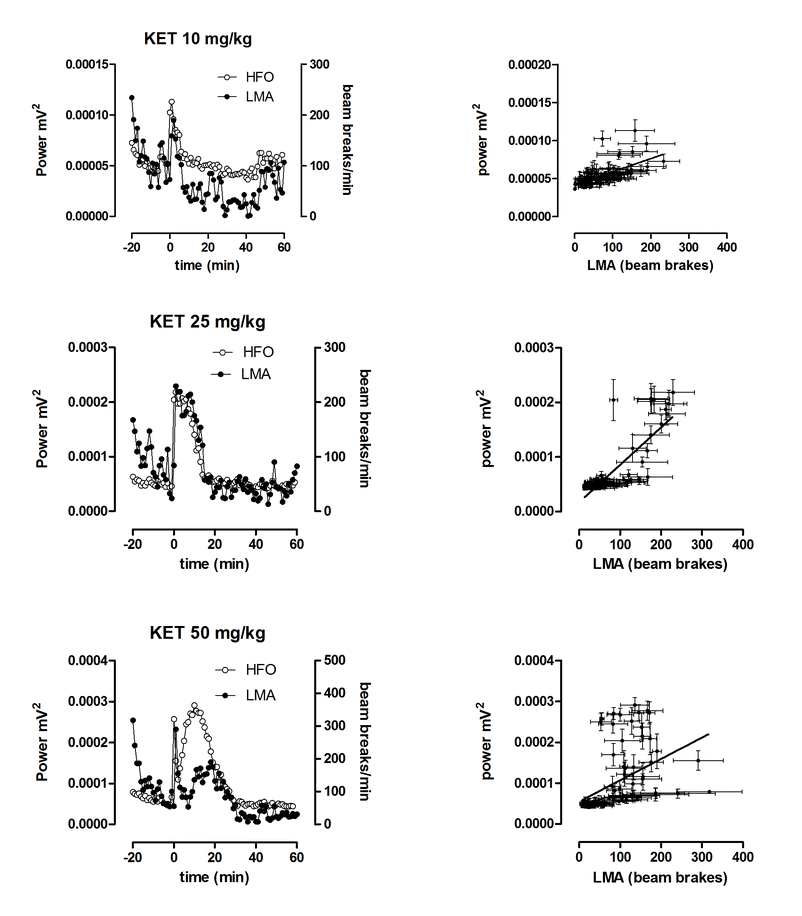

Supplement: Supplementary file 1 — Correlation between changes in ketamine-induced locomotion and HFO power (GIF 76 kb) [file 213_2015_4073_Fig7_ESM.gif]

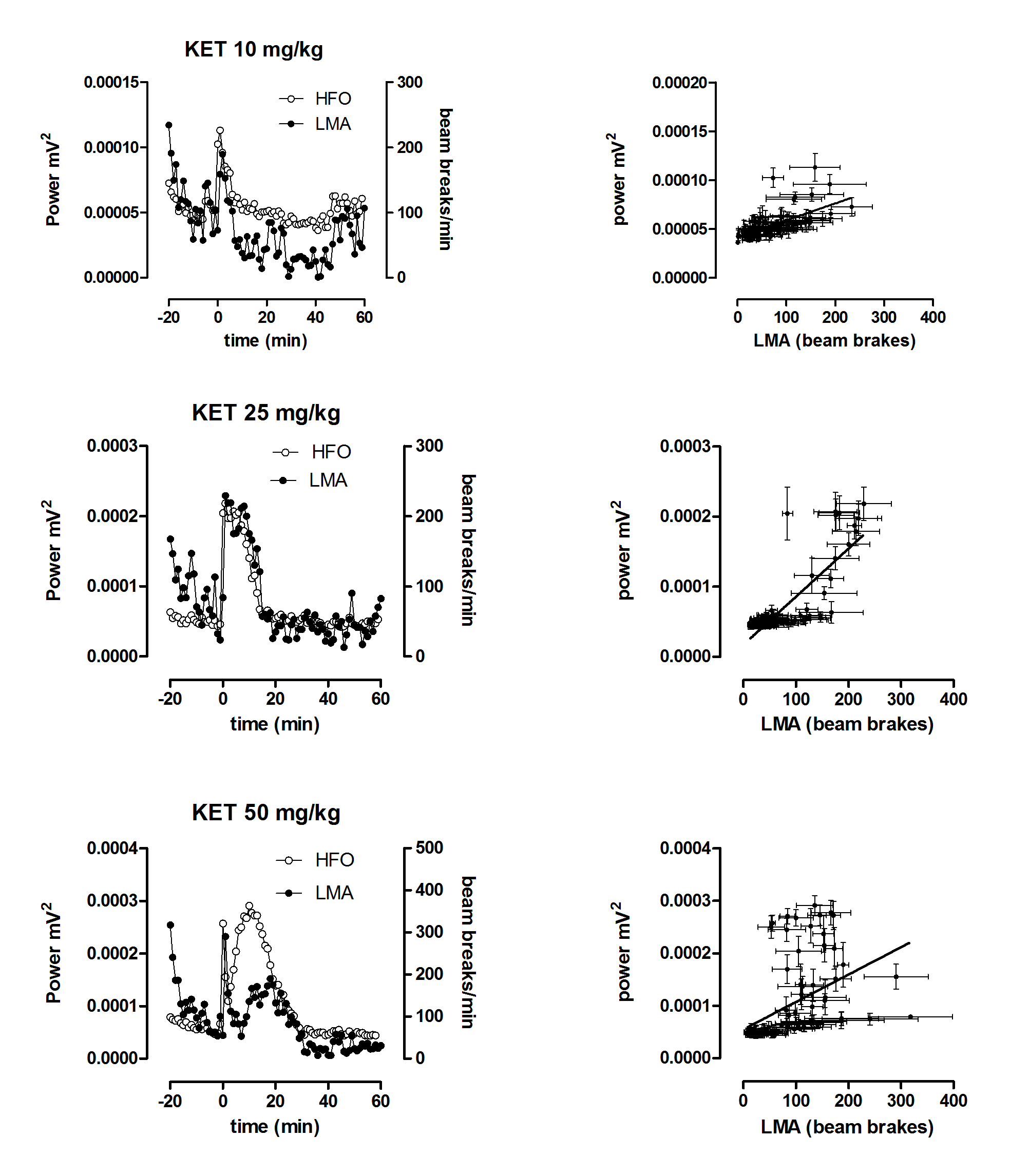

Supplement: Supplementary file 2 — High-resolution image (TIFF 447 kb) [file 213_2015_4073_MOESM1_ESM.tif]

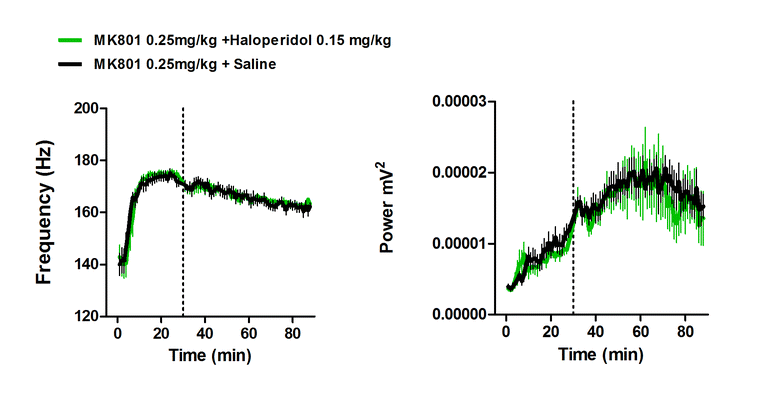

Supplement: Supplementary file 3 — Frequency and power of MK801-enhanced HFO after injection of 0.15 mg/kg haloperidol or vehicle (GIF 33 kb) [file 213_2015_4073_Fig8_ESM.gif]

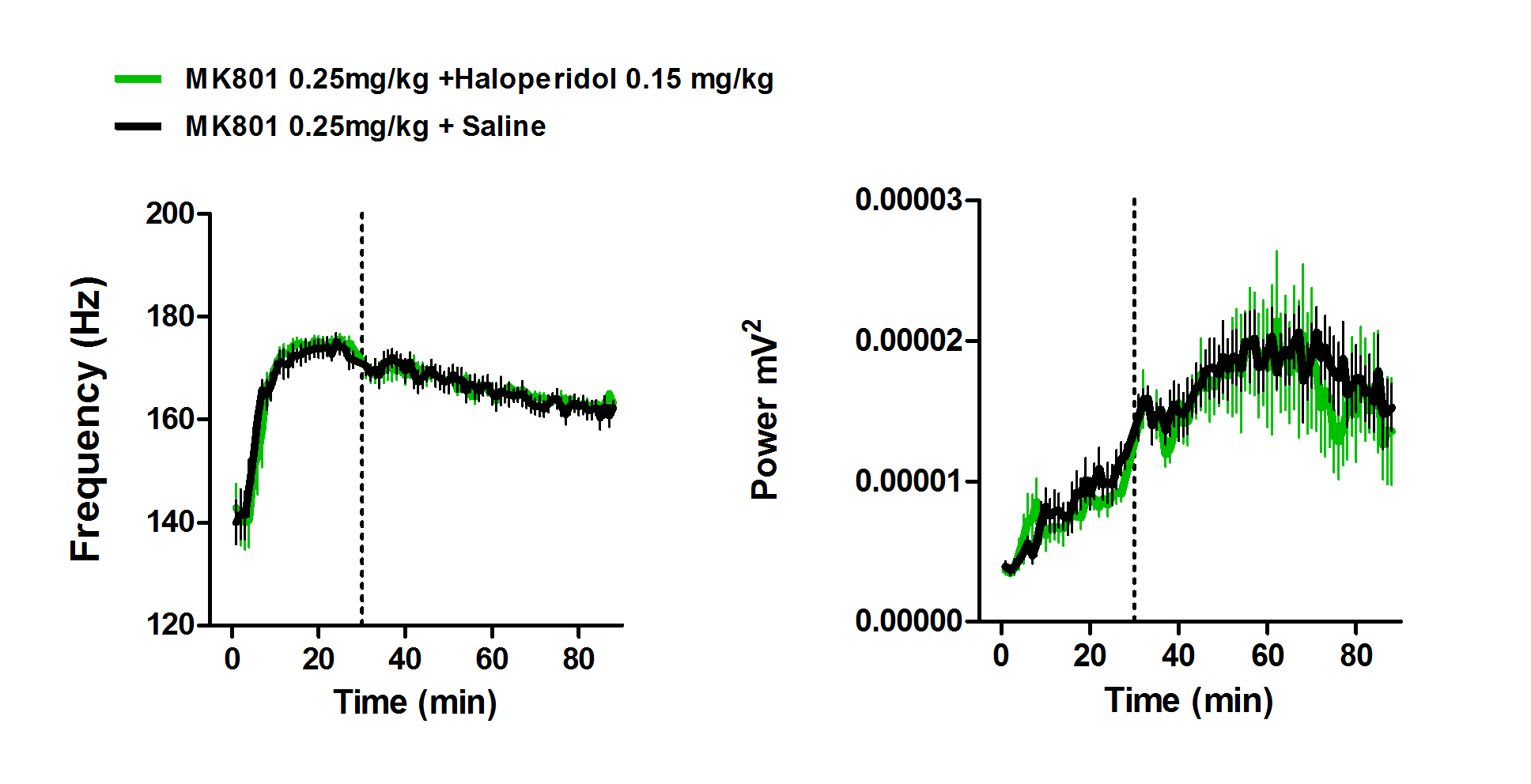

Supplement: Supplementary file 4 — High-resolution image (TIFF 238 kb) [file 213_2015_4073_MOESM2_ESM.tif]
