# Supplementary material for: Effects of NMDA receptor antagonists and antipsychotics on high frequency oscillations recorded in the nucleus accumbens of freely moving mice
Source: Psychopharmacology (Berl). 2015 Oct 8;232(24):4525–35. doi: 10.1007/s00213-015-4073-0 (PMC4646921; doi:10.1007/s00213-015-4073-0)
Supplement: Supplementary file 5 — Summary of the experimental groups used in the study (DOC 27 kb) [file 213_2015_4073_MOESM3_ESM.doc]

| Group 1 (n=4) | Group 2 (n=5) | Group 3 (n=6) | Group 4 (n=7) | Group 5 (n=6) | Group 6 (n=5) | Group 7 (n=6) |
| --- | --- | --- | --- | --- | --- | --- |
| Ketamine  (10, 25, 50 mg/kg) | MK801  (0.05, 0.1, 0.25, 0.5 mg/kg) | MK801 (0.25 mg/kg)  +  Clozapine  (1, 5, 15 mg/kg) | MK801 (0.25 mg/kg)  +  Glycine (2g/kg)/  NMDA (75 mg/kg) | MK801 (0.25 mg/kg)  +  8-OH-DPAT (1 mg/kg)/  MDL 11,939 (1 mg/kg) | MK801 (0.25 mg/kg)  +  Haloperidol (0.15 mg/kg) | MK801 (0.25 mg/kg)  +  SB269970 (1 mg/kg)/  BF2649 (5 mg/kg) |
|  |  |  | MK801 (0.25 mg/kg) + Haloperidol (1.5 mg/kg) | |  |  |
